# Supplementary material for: Controlling invasive rodents via synthetic gene drive and the role of polyandry
Source: Proc Biol Sci. 2019 Aug 21;286(1909):20190852. doi: 10.1098/rspb.2019.0852 (PMC6732378; doi:10.1098/rspb.2019.0852)
Supplement: Supplementary Material [file rspb20190852supp1.pdf]

Supplementary Information

Controlling Invasive Rodents via Synthetic Gene Drive and the Role of Polyandry

Andri Manser[1,*], Stephen J. Cornell[1], Andreas Sutter[2], Dimitri V. Blondel[3], Megan Serr[3], John Godwin[3], Tom A. R. Price[1]

[1] Institute of Integrative Biology, University of Liverpool, Biosciences Building, Liverpool, United Kingdom

[2] Centre for Ecology, Evolution and Conservation, University of East Anglia, Norwich, United Kingdom

[3] Department of Biological Sciences, North Carolina State University, Raleigh, NC 27695-7617, USA

[*] Andri.Manser@liverpool.ac.uk

Text S1 Polyandry and Sperm Competition with an Arbitrary Number of Mating Partners

Polyandry Let $f_{n,k}$ denote the frequency of a female mating with n males of which k are drive males. To calculate $f_{n,k}$, we need to know the probability that a female mates with n mating partners (where $n \in \{1 \dots n_{max}\}$), which we determine with parameter ϕ_n (with $\sum_1^n \phi_n = 1$). We assume here that the mating process is random. Because we only have two types of (fertile) males, the probability that a female encounters k *t-Sry* males in her sample of n mating partners follows a binomial distribution. For a given mating combination n, k, we thus have

$$f_{n,k} = \phi_n \binom{n}{k} y^k (1-y)^{n-k}. \qquad \text{(S1)}$$

For example, the probability that a female encounters two drive males ($k = 2$) if she mates with three males overall ($n = 3$) will be $f_{3,2} = \phi_n 3y^2(1-y)$.

Sperm Competition and Gene Drive The *genotypic outcome* of a given mating combination n, k will depend on the probability of fertilization by a t sperm, denoted by $p_{n,k}$. It depends on the level of gene drive s (where $s = 0.5$ denotes Mendelian inheritance and $s = 1$ represents complete drive) and sperm competitiveness r (see below). w/t and w/w males contribute the fraction $\frac{k}{n}$ and $\frac{n-k}{n}$, respectively, to the sperm pool (viable and non-viable). However, we assume that only r of a $D_w D_{\mathrm{Sry}}$ male's sperm is viable, of which a fraction s carries the driver. Parameter r hence defines $D_w D_{\mathrm{Sry}}$male sperm competitiveness relative to $D_w D_w$ wildtype males (whose sperm competitiveness equals unity). It is thus a measure of sperm precedence at fertilisation, also referred to as the loading of the sperm raffle. If all viable sperm have an equal fertilization probability, $p_{n,k}$ will be the fraction of viable *t-Sry* sperm $\frac{k}{n}sr$ divided by the total number of sperm $(1-k)+kr$. We thus have

$$p_{n,k} = \frac{kdr}{n-k+kr}. \qquad \text{(S2)}$$

If there are only drive males in a given male sample, $p_{n,k=n} = s$. Likewise, if all males are wildtype males, $p_{n,k=0} = 0$. Hence, a drive male's reduced sperm competitiveness (r) is only relevant if males of both genotypes are in the sample ($0 < k < n$ for $n > 1$). We can now calculate p, defined as the probability that a given ova is fertilised by *t-Sry* sperm over all possible matings, which is simply sum of the probability of D_t fertilisation of a particular mating $p_{n,k}$ weighed by possible mating

combinations $f_{n,k}$:

$$p_{n_{max}} = \sum_{n=1}^{n_{max}} \sum_{k=0}^{n} f_{n,k} p_{n,k}. \tag{S3}$$

For the polyandry model used in the main text, we consider a simple scenario where females either mate with one male (at frequency $1-\psi$) or two males (at frequency ψ). We can recover p_1 and p_2 from the main text by solving equations S1–S3 for $n_{max} = 2$, $\phi_1 = 1-\psi$, and $\phi_2 = \psi$.

Figure S1 illustrates how the number of mating partners n_{max}, drive male frequency in the population y, and drive male sperm competitiveness r affect the probability of drive fertilisation p.

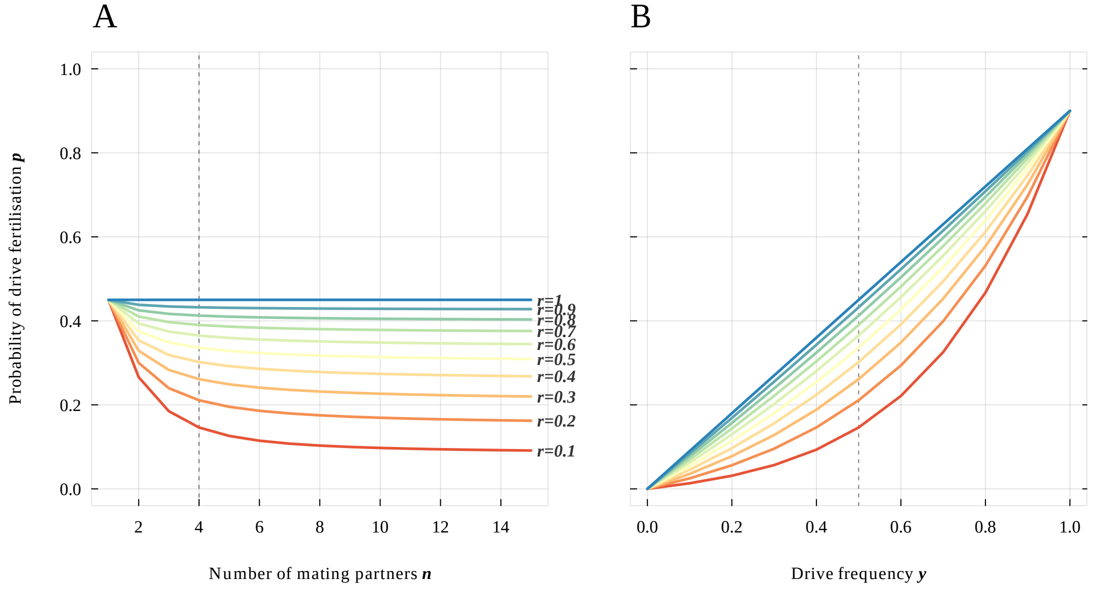

Figure S1. Polyandry and sperm competition reduce the probability that a female is fertilised by drive sperm. A. Polyandry is more effective if the number of mating partners n is high, although the marginal reduction in p gets smaller with every additional mating partner n. The different coloured lines represent varying levels of drive male sperm competitiveness r (colour gradient). The frequency of drive males in the population equals $y = 0.5$. B. Polyandry is less effective if the frequency of drive males is increased, as sperm competition is only relevant if drive males compete against wildtype males. The colour gradient again represents different levels of drive male sperm competitiveness r

Text S2 Analytical Results — Single Release

In the case of a single release ($\mu = 0$), our system of differential equations **??** simplifies to

$$\frac{dN}{dt} = bW - (m_1 + m_2 N)N$$
$$\frac{dW}{dt} = bW\frac{1-p}{2} - (m_1 + m_2 N)W \tag{S4}$$
$$\frac{dD}{dt} = bW\frac{p}{2} - (m_1 + m_2 N)D$$

where $p = s\frac{D}{W+D}$ under monandry.

Fixed points The system S4 will reach a longterm equilibrium $\{\hat{N}, \hat{W}, \hat{D}\}$ if $\frac{dN}{dt} = \frac{dW}{dt} = \frac{dD}{dt} = 0$. We have the following three solutions

$$\hat{N} = \frac{\frac{b}{2} - m_1}{m_2}, \quad \hat{W} = \frac{\frac{b}{2} - m_1}{2m_2}, \quad \hat{D} = 0$$
$$\hat{N} = 0, \quad \hat{W} = 0, \quad \hat{D} = 0 \tag{S5}$$
$$\hat{N} = -\frac{m_1}{m_2}, \quad \hat{W} = 0, \quad \hat{D} = D$$

of which only the first two are biologically feasible, as the third equilibrium requires negative mortality rates to produce $\hat{N} > 0$. The first equilibrium point describes a population where the driver is absent and the population is at carrying capacity. The second equilibrium corresponds to the trivial case where the population is not sustainable in the first place.

Stability The first equilibrium has the following three stability eigenvalues: $r_1 = -\frac{b}{2}, r_2 = m_1 - \frac{b}{2}, r_2 = -\frac{b}{2}(1-s)$. As expected, the population is viable if birth rates outweigh baseline death rates ($m_1 < \frac{b}{2}$). Moreover, and as mentioned in the main text, we can have $r_3 = 0$ as the leading eigenvalue when drive is complete ($s = 1$), which will change the dynamics of the system altogether, as the driver can be maintained stably in the population.

Text S3 Analytical Results — Continued Release under Monandry

To simplify equilibrium calculations under continued release ($\mu > 0$), we rearrange the system slightly. Let D' denote the number of drive carrying 'females' $XYwt$ in the population. We can now write the system as the following three differential equations

$$\begin{aligned} \frac{dW}{dt} &= bW\frac{1-p}{2} - (m_1 + m_2N)\,W \\ \frac{dD}{dt} &= bW\frac{p}{2} - (m_1 + m_2N)\,D + \mu \\ \frac{dD'}{dt} &= bW\frac{p}{2} - (m_1 + m_2N)\,D' \end{aligned} \tag{S6}$$

where the total population is now given as $N = 2W + D + D'$. We again search for equilibria by setting $\frac{dW}{dt} = \frac{dD}{dt} = \frac{dD'}{dt} = 0$. In the case of monandry, we get two biologically feasible solutions:

$$\begin{aligned} \hat{D}_1 &= \frac{-m_1 + \sqrt{m_1^2 + 4m_2\mu}}{2m_2},\ \hat{W}_1 = 0,\ \hat{D}'_1 = 0 \\ \hat{D}_2 &= \frac{2\mu}{b(1-s)}, \hat{W}_2 = \text{complicated expression}^\star, \Delta\hat{D}_2 = \text{complicated expression}^\star \end{aligned} \tag{S7}$$

*available on supplementary Maple file.

The eradication equilibrium and its stability As in the case a single release, the first equilibrium $\{\hat{W}_1, \hat{D}_1, \hat{D}'_1\}$ describes the (desired) outcome where the population is successfully eradicated ($\hat{W}_1 = 0$). Of the three eigenvalues of the Jacobian matrix, $r_{1,2,3}$, at the equilibrium point (see supplementary Maple file), only one can take on positive values ($r_1 = \frac{(1-s)b - m_1 - \sqrt{m_1^2 + 4m_2\mu}}{2}$). We can thus calculate the critical release rate $\mu_1^\star$ at which the eradication equilibrium becomes attracting as the point where the leading eigenvalues r_1 becomes negative ($r_1 < 0$ if $\mu > \mu_1^\star$). In other words, if release rates exceed $\mu_1^\star$, wildtype animals can no longer invade a population. Solving $r_1 = 0$ and replacing m_2 with $\frac{\frac{b}{2} - m_1}{K}$ (see Text S2), we have

$$\mu_1^\star = \frac{K\frac{b}{2}(1-s)\ \left((1-s)\frac{b}{2} - m_1\right)}{4R}, \tag{S8}$$

where R denotes the baseline reproductive rate $R = \frac{b}{2} - m_1$. Also note that $\mu_1^\star$ is a linear function of carrying capacity K, the standardised release rate $\mu_{\text{std},1}^\star = \frac{\mu_1}{K}$ will hence be independent of population size K.

The internal equilibrium and its stability The second, internal equilibrium corresponds to the case where both drive and wildtype individuals stably coexist. Although solutions for $\hat{W}_2$ and $\hat{D}'_2$ exist, they are unwieldy and offer little insight. However, note that the dynamics of drive males D and 'females' D' only differ by the fact that males are released into the population while drive 'females' D' are not. More formally, at equilibrium, we have $\Delta\hat{D} = \hat{D} - \hat{D}' = \frac{\mu}{m_1+m_2N}$. Together with the fact that D' cannot take on negative values, this implies that $0 \leq \hat{D}' \leq \hat{D}$ for biologically relevant parameter values ($\mu, m_1, m_2 \geq 0$). As a plausible approximation, we can thus examine stability of the two boundaries where $\hat{D}' = 0$ or $\hat{D}' = D$. Note that this argument is mathematically not strictly correct, as $0 \leq \hat{D}' \leq \hat{D}$ does not guarantee that the stability eigenvalues or critical release thresholds are also sandwiched between those two cases (because they may depend non-monotonically on D'). They may nevertheless serve as a useful, plausible approximation.

- In the first boundary case where $D' = 0$, our differential equation system reduces to

$$\begin{aligned} \frac{dW}{dt} &= bW\frac{1-p}{2} - (m_1 + m_2N)\,W \\ \frac{dD}{dt} &= bW\frac{p}{2} - (m_1 + m_2N)\,D + \mu \end{aligned} \tag{S9}$$

  where, importantly, $N = 2W + D$ (since $D' = 0$). System S9 is much easier to handle, and allows us to calculate the two eigenvalues of the Jacobian matrix at the internal equilibrium (see Supplementary Maple file). Once again, we can calculate critical release rate $\mu_2^\star$ where the equilibrium becomes unstable and the population collapses. We have

$$\mu_2^\star = \frac{K\frac{b}{2}(1-s)\left(2bs - R - \sqrt{4bs(2bs-R)}\right)}{R}, \tag{S10}$$

  with $R = \frac{b}{2} - m_1$. Note that $\mu_2^\star$ is again independent of carrying capacity K when standardised $\mu_{\text{std},2}^\star = \frac{\mu_2}{K}$.

- In the second boundary case where $D' = D$ we have system S9 with the important difference that $N = 2W + 2D$ (since $D' = D$). We can again calculate the critical release rate $\mu_3^\star$ using the approach used above (Supplementary Maple file). We have

$$\mu_3^\star = \frac{KR(1-s)}{8s}. \tag{S11}$$

Testing approximate solutions To assess the accuracy of the two approximate solutions, we compared them against numerical solutions based on the full equation system (see Figure S2). For all parameter combinations considered, the two analytical approximations $\mu_2^\star$, $\mu_3^\star$ predict numerically calculated release thresholds relatively accurately. Numerically calculated release thresholds were indeed placed inside the interval of the two boundary cases $[\mu_2^\star, \mu_3^\star]$. Moreover, the critical release rate for the eradication equilibrium $\mu_1^\star$ was smaller than $\mu^\star$ for the internal equilibrium for all parameter values considered ($\mu_1^\star < \mu_3^\star < \mu_2^\star$). Hence, at least for the parameter space examined, we can distinguish three qualitatively different dynamics as release rates of drive animals μ into the target population are increased.

1. If release rates μ are sufficiently low such that $\mu < \mu_1^\star$, the internal equilibrium is stable and the eradication equilibrium unstable. The resident population is sustainable.

2. At intermediate release rates where $\mu_1^\star < \mu < \mu_3^\star$ we have a bistable system where both the internal and the eradication equilibrium are stable. Note that the existence of bistability also implies that examining the stability criteria of the eradication equilibrium (invasion criteria), which is mathematically straightforward, is *not* sufficient to predict population eradication.

3. If release rates are sufficiently large such that $\mu > \mu_2^\star$ the internal equilibrium is repellent and eradication is stable. The population collapses. Note that, because we had $\mu_1^\star < \mu_2^\star$ for all combinations considered, the condition $\mu > \mu_2^\star$ was both a sufficient and necessary for a successful eradication.

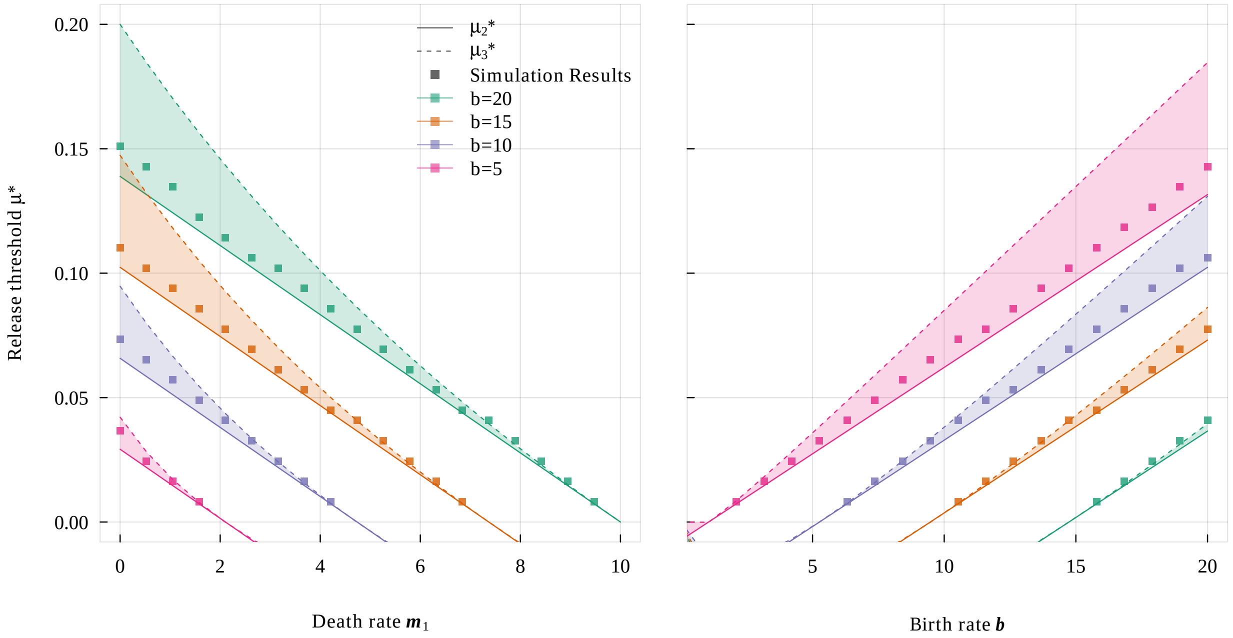

Figure S2. The critical release effort $\mu^{\star}_{\text{std}}$ required to push a population to eradication as a function of birthrate b and death rate m_1 based on analytical approximation (solid and dotted line for $\mu^{\star}_2$ and $\mu^{\star}_3$, respectively) and numerical calculation (squared dots). Drive strength in both panels is $s = 0.9$.

Text S4 Differential survival and mate choice

In this section, we analyse the effect of differential survival rates between genotypes and precopulatory mate choice on a) the success of *t-Sry* release campaign and b) on the strength of the polyandry effect explored in the main text. Because all four genotypes now have different fitness, we have to track them separately. We have the following equation system

$$\begin{aligned}
\frac{dW_X}{dt} &= bW_X\frac{1-p}{2} - (m_1 + m_2 N)\, W_X \\
\frac{dW_Y}{dt} &= bW_X\frac{1-p}{2} - (1+s_m)\,(m_1 + m_2 N)\, W_Y \\
\frac{dD_X}{dt} &= bW_X\frac{p}{2} - (1+s_m+s_t)\,(m_1 + m_2 N)\, D_X \\
\frac{dD_Y}{dt} &= bW_X\frac{p}{2} - (1+s_m+s_t+s_{2\mathrm{Sry}})\,(m_1 + m_2 N)\, D_Y + \mu.
\end{aligned} \tag{S12}$$

Population size N is again given as the sum of all four genotypes: $N = W_X + W_Y + D_X + D_Y$.

Differential survival Here, s_i represent survival differences between the different genotypes. They quantify the survival cost (or benefit if $s_i < 0$) of being (phenotypically) male (s_m), carrying the t haplotype (s_t), and the cost of carrying an extra copy of the *Sry* ($s_{2\mathrm{Sry}}$).

The top panels in Figure S3 illustrate the effect of t related survival costs s_t on the required release effort to eradicate the population $\mu^\star_{\mathrm{std}}$, and how that relationship is affected by polyandry levels ψ (the primary focus of this manuscript). As expected, required release efforts increase with increasing survival costs of the driver. Interestingly, the independent effects of survival (moving horizontally on upper panels in Fig. S3) and polyandry (moving vertically on upper panels in Fig. S3) are nearly identical, at least for the parameter combinations considered. Additionally, it appears that the two effects are close to additive.

In Figure S4, we investigate the impact of a survival cost on being phenotypically male (s_m) and of carrying an extra copy of the *Sry* gene $s_{2\mathrm{Sry}}$ on release dynamics. s_m has only very small effects on required release rates $\mu^\star_{\mathrm{std}}$. This is because the success of the drive construct *mainly* depends on how competitive *t-Sry* males are *relative* to wildtype males, which is independent of overall male survival. In fact, lower male survival in some cases even decreases the required release effort because it creates decreased (density-dependent) competition on reproductive females. The

fact that the dynamics are dominated by the fitness contrast between drive and wildtype males also explains why the cost of carrying an extra *Sry* $s_{2\text{Sry}}$ is nearly identical to s_t (as the survival difference between drive and wildtype males is $s_t + s_{2\text{Sry}}$, as reflected in the symmetry in right panel in Figure S4 with respect to the diagonal).

Mate choice We further explore the possibility that females have a precopulatory mating preference (preexisting or evolved), most likely to avoid drive carrying males. Let α measure the fixed relative strength of females to avoid matings with drive males. The probability of mating with a drive male f_t is given as

$$f_t = y\frac{1-\alpha}{1-\alpha y}. \tag{S13}$$

where the denominator $y(1-\alpha)+(1-y) = 1-\alpha y$ is a normalising constant that ensures that the probabilities of the two possible matings (W and D) add up to 1. Hence, females avoid drive males in the spectrum where $0 < \alpha < 1$, $\alpha = 0$ represents random mating, and $\alpha < 0$ would denote a preference for drive males. Substituting f_t for y in Equation 2, we then have

$$p_2 = f_t s\left(1 - \psi(1-f_t)\frac{1-r}{1+r}\right). \tag{S14}$$

The lower three panels in Fig. S3 show the the effects of mate choice on required release efforts. Again, as expected, more animals need to be released if resident females avoid drive males. Note that mate avoidance becomes particularly problematic from a intervention perspective if drive male avoidance is strong ($\alpha > 0.6$). Again, there are no clear interactive effects with polyandry ψ.

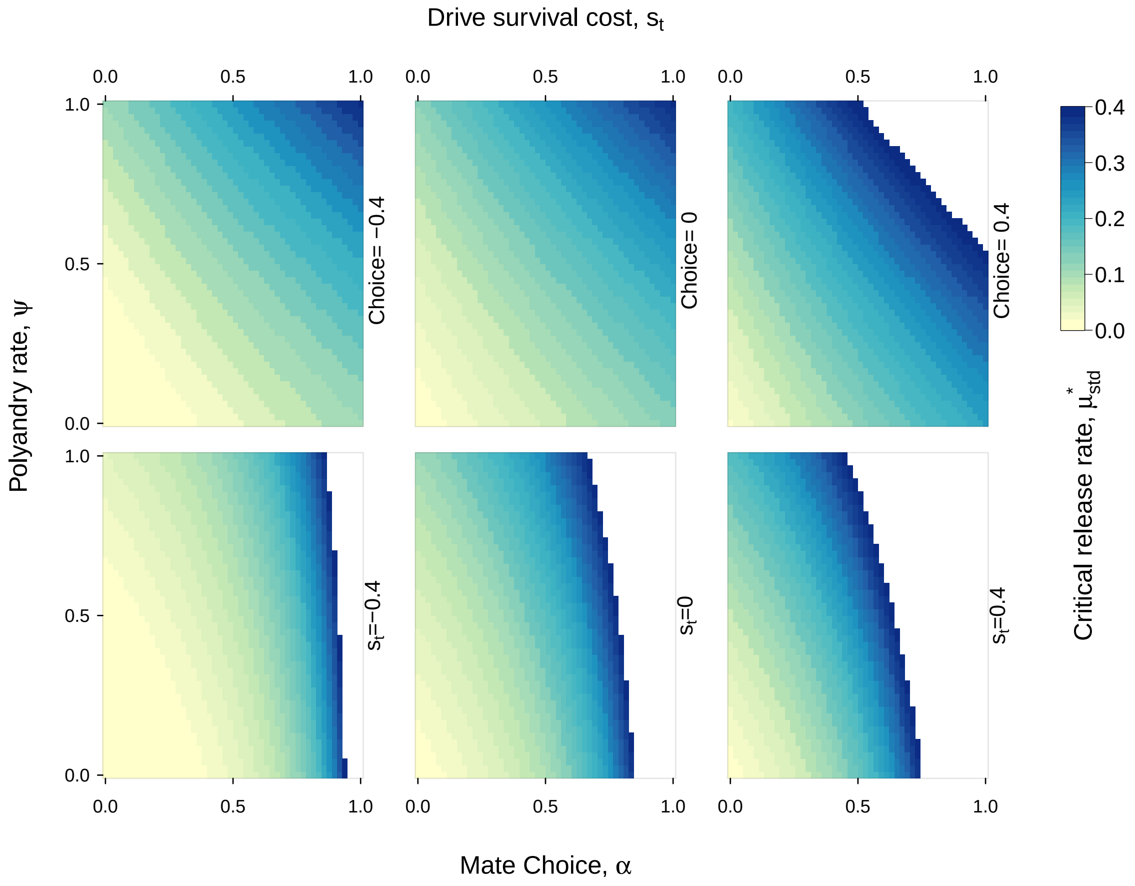

Figure S3. The critical release effort $\mu^{\star}_{std}$ required to eradicate a population as a function of the survival cost of the driver s_t, mate choice against the driver α, and polyandry rate ψ. The three upper panels focus on drive survival costs s_t for three different levels of mate choice ($\alpha = -0.4, 0, 0.4$). The three lower panels focus on mate choice for three levels of drive survival cost ($c_t = -0.4, 0, 0.4$). The white area represent parameter combination where the required release rate exceeded the range considered ($\mu^{\star}_{std} > 0.4$). Remaining parameter values for all panels: $s = 0.9$, $r = 0.2$, $s_m = 0$, $s_{2\mathrm{Sry}} = 0$, $b = 4$, $m_1 = 1$, $K = 1000$.

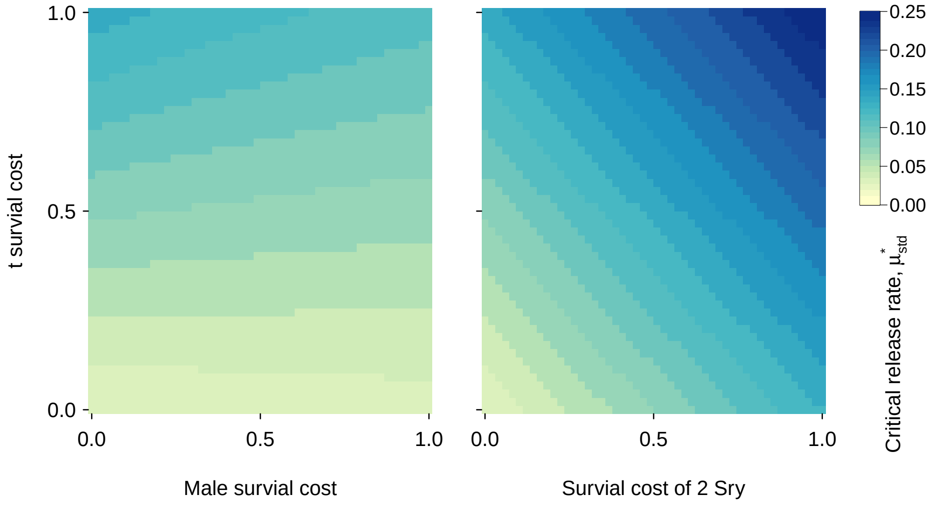

Figure S4. The critical release effort $\mu^{\star}_{std}$ required to eradicate a population as a function of the survival cost of the driver s_t, the survival cost of being (phenotypically male) s_m, and the cost of carrying an extra copy of the *Sry* gene. Remaining parameter values for all panels: $s = 0.9$, $r = 0.2$, $\psi = 0$, $\alpha = 0$, $b = 4$, $m_1 = 1$, $K = 1000$.

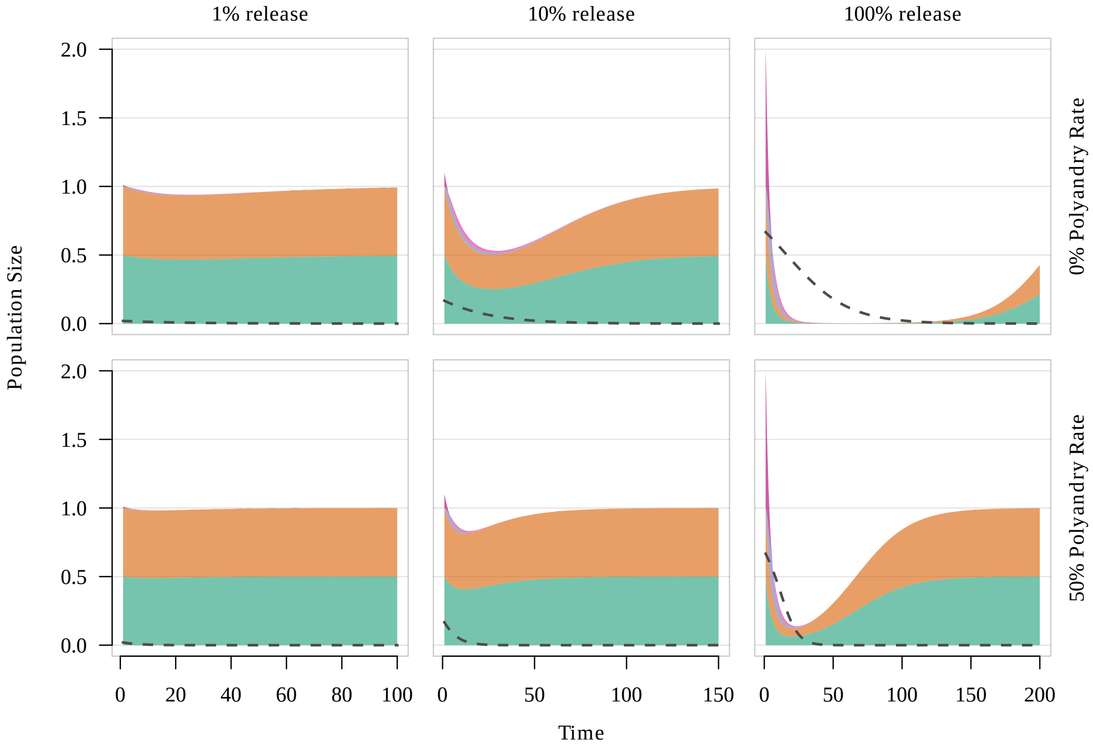

Figure S5. The number of wildtype males and females (W in green and orange), *t-Sry* males (D in violet) and *t-Sry* 'females' (pink) as a proportion of carrying capacity K after a single release of drive individuals into the population. The dotted line represents the drive frequency in the population. Columns represent different release efforts (number of males release as a proportion of carrying capacity K). The two rows depict a monandry ($\psi = 0$) and a polyandry ($\psi = 0.5$, $r = 0.2$) scenario, respectively. The driver cannot eradicate the population in any of the scenarios shown. Remaining parameter values: $s = 0.9$, $b = 0.9$, $m_1 = 0.4$, $K = 1000$
